# Prioritized Degree Distribution in Wireless Sensor Networks with a Network Coded Data Collection Method

**DOI:** 10.3390/s121217128

**Published:** 2012-12-12

**Authors:** Jan Wan, Naixue Xiong, Wei Zhang, Qinchao Zhang, Zheng Wan

**Affiliations:** 1 School of Computer Science and Technology, Hangzhou Dianzi University, 1# of NO.2 street, Xiasha Higher Education District, Hangzhou 310037, Zhejiang, China; E-Mails: wanjian@hdu.edu.cn (J.W.); xiongnaixue@gmail.com (N.X.); qinchaoz@163.com (Q.Z.);; 2 School of Information Technology, Jiangxi University of Finance and Economics, No.632 West Fenglin Street, Nanchang 330013, Jiangxi, China; E-Mail: cloudcity66@yahoo.com.cn

**Keywords:** wireless sensor networks, distributed storage, network coding

## Abstract

The reliability of wireless sensor networks (WSNs) can be greatly affected by failures of sensor nodes due to energy exhaustion or the influence of brutal external environment conditions. Such failures seriously affect the data persistence and collection efficiency. Strategies based on network coding technology for WSNs such as LTCDS can improve the data persistence without mass redundancy. However, due to the bad intermediate performance of LTCDS, a serious ‘cliff effect’ may appear during the decoding period, and source data are hard to recover from sink nodes before sufficient encoded packets are collected. In this paper, the influence of coding degree distribution strategy on the ‘cliff effect’ is observed and the prioritized data storage and dissemination algorithm PLTD-ALPHA is presented to achieve better data persistence and recovering performance. With PLTD-ALPHA, the data in sensor network nodes present a trend that their degree distribution increases along with the degree level predefined, and the persistent data packets can be submitted to the sink node according to its degree in order. Finally, the performance of PLTD-ALPHA is evaluated and experiment results show that PLTD-ALPHA can greatly improve the data collection performance and decoding efficiency, while data persistence is not notably affected.

## 1. Introduction

**Figure 1.**
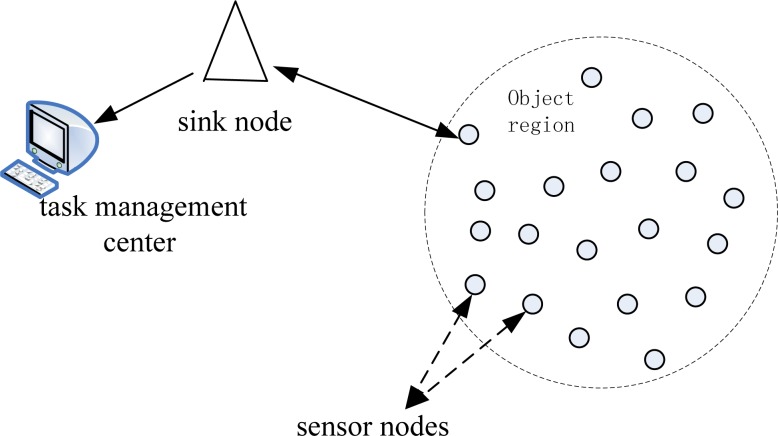
Typical architecture of a wireless sensor network.

Wireless *ad-hoc* networks are usually characterized by self-organizing, multi-hop, dynamic topology and energy-resource restrictions. One typical application of *ad-hoc* networks is a wireless sensor network (WSN), which can be employed in emergency or disaster scenarios. WSNs usually consist of a large number of low-cost nodes randomly deployed in a certain monitoring region. These nodes work together to obtain data about the environment (as seen in Figure 1). Sensor nodes are usually equipped with limited energy, computing and communication resources, and the communication between sensor nodes is usually unreliable. The sensor nodes in a WSN system can be employed in areas where it is dangerous for human involvement, to monitor objects, detect fires or other disaster scenarios [1].

The availability of WSNs can be greatly affected [2], since the sensor nodes in WSNs are vulnerable to the brutal external environment conditions. On the one hand, all kinds of unpredictable events may lead to failures of sensor nodes, which may cause the potential loss of source packets collected or transferred in the network. On the other hand, the sensed data in disaster scenarios are usually required to be collected as quickly as possible, but the speed of data generation in a large scale sensor network usually greatly exceeds the bandwidth capacity of the delivery channel to the sink node, so that it may takes a long time to collect all data in the network. Therefore, it is important to find a mechanism to increase the decoding and collection efficiency, while the sensed data can be distributed in the damaged network with high robustness. Such mechanism is expected to increase the persistence and reliability of the important data in sensor networks.

The simple backup mechanism is typical method to improve data persistence of distributed information systems [3], but such mechanism shall cause serious data redundancy which greatly affects the efficiency of the network [4]. Furthermore, considering the uncertainty of the fault type and location of sensor nodes, simple backup mechanism can hardly prevent the object data and its simple backup from been destroyed at the same time.

The network coding theory by Ahlswede *et al.*[5] was proposed based on the principle of information theory. Network coding theory allows intermediate relay nodes to recombine the data packets message before forwarding it, so that the messages of several packets can be partly implied in one coded packet. Such coded packets can be randomly disseminated into the WSN systems, so, when one source node fails, its data can still survive with high probability elsewhere in the network. When the system tries to recover the coded data, after forwarding the coded data packets to the destination node with appropriate order, the source data can be easily obtained by decoding with low communication cost. By this means, the data storage and collection strategy based on network coding [3] can effectively avoid the mass redundancy of simple backup strategies, improving the data persistence and transmission efficiency. Aly *et al.*[6] proposed an LT Codes-based distributed storage algorithm (LTCDS), a persistent data storage solutions with low data redundancy [7]. LTCDS can effectively tolerate the failure of WSN nodes without geographic or routing information.

However, due to the bad intermediate performance of LTCDS [7], a serious ‘cliff effect’ may appear during the decoding period [8], that is to say, source data are hard to recover from sink nodes before sufficient encoded packets are received. Consequently, in this paper, an effective data collection mechanism based on network coding is to be designed to improve the data collection performance and decoding efficiency of a WSN system. Generally, the research environment shall be defined as a wireless sensor network with *n* nodes of limited storage and computing resources. Among these nodes, *k* nodes perform as real sensors which generate source data (*k* < *n*), and one mobile data collector can be deployed into the network when the data collection period begins, which visits the sensor nodes according to a certain access path and retrieves the source data. In order to improve the decoding efficiency of the data recovery process, in this paper, the principle of ‘cliff effect’ for network coding strategies is observed in this paper, and a LT Codes-based prioritized distributed data storage and dissemination algorithm PLTD-ALPHA is presented to achieve better data persistence and recovery performance. With PLTD-ALPHA, prioritized degree distribution is designed to arrange the data in sensor network nodes to present a degree distribution trend that they are distributed according to the degree level predefined in the initiation period of the network, from low to high. Therefore, when the mobile collector begins to gather coded data packets, it can first accumulate low-degree codewords which are easy to decode, and the high-degree codewords shall be collected later when enough decoding factors are obtained by the collector. In this way, the data collection performance and decoding efficiency can be greatly improved while data persistence is assured.

This paper is organized as follows: in Section 2, some previous related works in this area are presented and discussed. Then, in Section 3, the network settings are introduced and the problem scenario is formulated. In Section 4, the details of the design of PLTD-ALPHA are described. Section 5 presents several simulation results and performance analysis. Finally, Section 6 presents some conclusions and discusses future works.

## 2. Related Works

The data collection mechanism of a WSN is relevant to the distributed storage mechanism of the WSN to a great degree. Typical traditional WSN data collection mechanisms were designed based on routing models, such as the Sensor Protocols for Information via Negotiation (SPIN) [9], Directed Diffusion (DD) [10], and Low Energy Adaptive Clustering Hierarchy (LEACH) [11] protocols. However, due to the characteristics of wireless sensor networks, there are a lot of problems for routing models in WSN systems such as the controlling of dynamic topology and the “routing hole” problem [12]. These problems can seriously affect the performance of WSN systems, especially in disaster scenarios, since such WSN system presents highly dynamic characteristics, and the network topology has to constantly change according to the availability of sensor nodes. On the other hand, in a routing model, some key nodes may have to continuously undertake heavy communication tasks while other nodes have few opportunities to forward messages, which can affect the balance of energy consumption in WSN and shorten the life cycle of the whole system. Generally, routing models are impractical for resource constrained WSN systems.

A network coding model with random data exchanging could be an alternative solution. In the recent years, various algorithms have been proposed to solve distributed storage and collection problems with network coding, such as Reed-Solomon codes [13], LDPC codes [14], LT codes [15] and so on. Ahlswede *et al.* determined the multicast capacity in a network of lossless links and showed that achieving the multicast capacity requires in general the use of network coding [5]. Network coding theory allows intermediate relay nodes to recombine the data packets message before forwarding it. When one source node fails, its data can still survive elsewhere in the network with high probability. The data storage and collection strategy based on network coding [3] can effectively avoid the mass redundancy of simple backup strategies, improving the data persistence and transmission efficiency. Li *et al*. proved that linear network coding is sufficient to achieve network multicast capacity [16], which established the foundation for the development and application of network coding theory.

The COPE coding method [17], proposed by Katti *et al.* based on the XOR operation, established the basic coding system to study the various characteristics of network coding in wireless sensor networks. With COPE, nodes encode source data packets according to their neighbor tables and the packet list to be sent before forwarding the coded packet. The collector node can recover the original packets by a simple exclusive-or operation. Kamran *et al.* analyzed persistence of data in wireless sensor networks and proposed the Growth Codes mechanism [18]. Growth Codes are designed for zero-configured WSN systems. Its degree of coding increases linearly while the packets recovered by the collector accumulate. A series of extensive researches have been proposed based on Growth Codes [19,20].

Network coding has been used for distributed storage and data collection in many network scenarios [21,22]. Xu, Fan *et al*. proposed a novel protocol called buffer-node based data collecting protocol (BNP), which shows novel performance in dense networks while it doesn’t reach the expected results in relatively sparse networks. Regulative Growth Codes [23] is a semi-random sequencing strategy for data exchanging instead of other completely random strategies, which introduces a self-detection mechanism to eliminate a large number of redundant exchanges. Xu, Fan *et al*. further introduce the modified BMNP for sparse WSN systems, which improves on the performance of BNP to some extent. The previous works employ a completely random method to select the neighbors and to exchange data, which inevitably affects the data transmission efficiency. On the other hand, such methods are usually unfair for the nodes far away from the sink node, since they have low probability to deliver their data to the sink node, especially in relatively sparse networks.

Fountain Codes were originally proposed only to satisfy the requirement of data broadcasting and distribution for mass data applications. A “digital fountain” allows any number of heterogeneous receivers to acquire content with optimal efficiency, and furthermore, no feedback channels are needed to ensure reliable delivery, even in a network with a high loss rate. For WSN systems, rateless distributed storage solutions can be designed based on Digital Fountain Codes. LT-codes are typical distributed Digital Fountain Codes, proposed by Luby in 2002 to offer a solution for the distribution of scalable and fault-tolerant data over computer networks. LT Codes are sparse random linear Fountain Codes with simple decoding algorithms, which greatly decrease the coding complexity. In 2006 Shokrollahi proposed an improved LT code, the “Raptor Code” [24]. Raptor codes can be regarded as an extension of LT-codes with a linear time encoding and decoding model. Raptor codes produce a potentially infinite stream of symbols such that any subset of symbols with the size of k*(1 + *epsiv*) is sufficient to recover the original *k* symbols with high probability. Both LT codes and Raptor codes are originally proposed based on the principle of LDPC codes, which are designed for the erase information channel. Such codes are more appropriate for applications in traditional computer networks rather than distributed *ad-hoc* networks (including WSNs).

In order to implement the idea of Digital Fountain Codes to adapt to the characteristic of WSN systems, Dimakis *et al.* proposed a decentralized Fountain Codes based on geographical routing [25]. With this mechanism, the storage nodes in the network collect data by randomly querying selected sensor nodes. However, such querying relies on routing to select data packets, and it argues that every node should know its own location information, so such a strategy is impractical for most WSN systems randomly deployed in harsh environments. The most relevant works for this paper are [26] and [6]. The concept of “random walk” model is adopted for the design of distributed storage mechanism in these works. In [26], Lin proposed a decentralize scheme to improve the data persistence and reliability. Source data packets randomly walk across the whole network and each node provides the number of random walks according to the number of storage nodes *n* and source nodes *k* in order to achieve the stationary distribution. The computational complexity of such storage strategy is very large, because there are large numbers of probabilistic forwarding matrixes to be calculated. Aly *et al.*[6] proposed a distributed scheme LTCDS based on LT codes, with a simple coding scheme and good data persistence performance. However, a serious ‘cliff effect’ may appear during the decoding period of LTCDS, which means that, source data are hard recover from the sink node before sufficient encoded packets are collected. Liang *et al.* presented a distributed coding scheme based on overhearing named LTSIDP [27]. By overhearing, each node can receive the information forwarded by their neighbors, LTSIDP increased the information utilization ratio without extra communication cost, but all sensor nodes are required to offer extra storage to maintain a forwarding list. Paper [28] proposed a distributed packet-centric rateless coding technique to solve the problem of data gathering. Packet-centric means that in the encoding phase, it just has to control the data packets itself, which can tolerate node failures. In [29], the above methods are observed in the special scenarios where sensor nodes are deployed in an inaccessible location. A simple edge detection method is utilized to find the surrounding nodes and the collector can recover source data by visiting these surrounding nodes.

The above distributed schemes studied the performance of data persistence, but they did not pay much attention to the decoding efficiency, and the ‘cliff effect’ during the decoding period is hard to overcome. Although Hagedorn *et al.*[30] tried a feedback mechanism to improve the decoding performance, but it costs vast communication resources. The methods in [8] and in [31] adjusted the coding sequence at the sender in order to maintain a high decoding ratio in a channel of high error environment, but they cannot be employed in distributed storage scenarios. This paper intends to propose a new distributed storage and collection strategy to improve data collection and decoding performance of WSNs, while data persistence is not notably affected.

## 3. Scenario and Network Model Definition

Before further illustration of our strategy, the problem scenario and network model description are introduced in this section. In this paper, the wireless sensor network can be considered as a distributed storage system. With a well-designed strategy, data packets from the original sensor nodes can be efficiently stored in many redundant storage nodes. Then, our research environment can be abstracted as a distributed storage system. In this system, the entire network is a huge data source, and the sensor nodes continuously generate data packets, while the collector node can be regarded as the container to collect encoded packets collected in the WSN system.

Distributed storage systems have been widely researched in classic network storage system studies. Suppose there are *k* nodes, each node generates one data packet, therein *n* memory nodes perform as the role of storage nodes. Theoretically, after the packet are disseminated into the network, all the *k* data packets can be recovered by querying the information from a random subset of the network with slightly more than *k* nodes. A similar model is adopted in this work, that is to say, if k*(1 + *ε*) data packets can be received from the surviving nodes, all the *k* source packets can be recovered. Since in wireless sensor networks, sensor nodes can fail extremely easily due to energy exhaustion or the impact of the external environment, and the failure points are completely unpredictable, such a model can offer a larger opportunity for the data in the sensor nodes to survive the random node failures.

**Figure 2.**
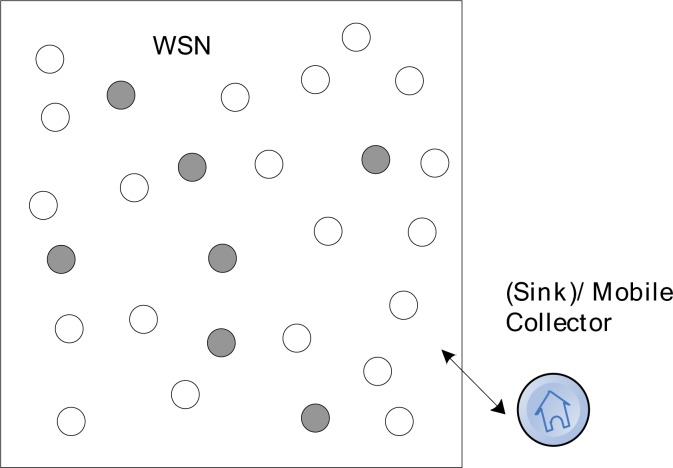
The network model.

The object system is a wireless sensor network consists of *n* redundant nodes with limited storage and computing resources. As shown in Figure 2, there are *n* sensor nodes randomly deployed in a region and a collector is placed at the entrance of the network, where black nodes are source nodes.

In this figure, the network consists of *n* nodes and a collector *S* which are uniformly distributed at random in a region of M*M. Each node has a unique ID. The network can be considered as a graph *G* = (*V*, *E*), where *V* = {*v*_1_, *v*_2,_ … *v*_n_} denote the set of nodes and *E* represents the set of links. Each node has a communication radius *r*. The *v*_i_, and *v*_j_ can be defined as neighbors if their distance *d*(*v*_i_, *v*_j_) < *r* and there exists a link between them, *i.e.*, (*u*, *v*) ∈ *E. S* is a mobile data collector, which gets into the network from predefined entrance, sequentially accesses the nodes it meets and retrieve the data packets stored in these nodes. There are several assumptions for this model:

1. All nodes are equivalent, the geographic locations of the network nodes are unknown and each node can only gather the information of its neighbor nodes within an appropriate communication range.
2. The source node set is *V*_s_. The nodes in *V*_s_ act not only as source nodes to generate sensed data packets, but also as storage nodes. The nodes in *V*_s_ are independently randomly distributed in the network, and each node does not maintain routing tables or network topology information.
3. The nodes in *V*_s_ only possesses limited local network information, such as the number of neighbors, The total number of nodes in the network *n,* and the number of source nodes *k*. They do not know other information such as the maximum neighbor degree for the nodes of the whole network.
4. The storage capacity of the nodes is quite limited and, under normal circumstances, only one packet can be stored in a node.

**Figure 3.**
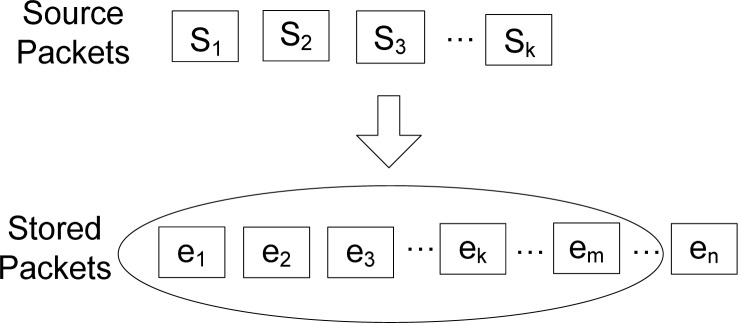
The distributed storage system.

Among these *n* nodes, there are *k* source nodes that monitor the environment and generate data. *V*_s_ is the set of source nodes, which generates source packets as well as been distributed randomly as storage nodes, and *k*/*n* is not very large. As shown in Figure 3, the *k* source packets shall be coded into a bigger packet set, which includes some necessary redundant data. By this means, the persistence of data and reliability of the network can be greatly enhanced. The nodes in this model don’t maintain a routing mechanism and they only possess restricted local network information.

A random walk model is adopted to disseminate the source data in the WSN system. Each sensor node randomly sends its data packet to its neighbor, so that all packets can be distributed to the entire network. The advantage of the random walk model is that the nodes in a WSN system only have to know the local information of its neighbors, rather than the global information of the network. On the other hand, the balance of energy consumption in WSN can be greatly improved with a random walk model, since all nodes in the system have fair opportunities to be chosen to encode and forward data packets.

## 4. PLTD-ALPHA Algorithm and Analysis

In this section, the background of Fountain Codes, especially, the LT codes [7], is to be introduced. Then we will propose PLTD-ALPHA, an optimized degree distribution strategy for persistent data storage and collection. PLTD-ALPHA is designed to help accelerate the decoding process for the persistent data in a sensor network.

### 4.1. Fountain Codes in Distributed Systems

Rateless erasure correcting codes [3] have the standout characteristic that, for a fixed number of information symbols, any number of code symbols can be generated, and the data can be recovered with high probability from any subset of the code symbols which is only slightly larger than the original data set itself. The information and code symbols are bit-strings of length *l*, and all encoding and decoding processes involve only bitwise XOR operations. Fountain Codes are typical rateless codes. The number of source blocks *d* employed to generate an encoded block is defined as the ‘coding degree’ for the Fountain Codes. Each encoded packet for Fountain Codes is obtained by XOR-ing *d* packets randomly and independently chosen from source packets, where *d* is drawn from a probability distribution. To recover the original information, *k* source blocks can be decoded from a subset of k*(1 + *ε*) encoded blocks, where *ε* > 0 is a small integer.

#### 4.1.1. LT Codes

LT Codes [7] are a special class of Fountain Codes, which are designed with Ideal Soliton or Robust Soliton distributions. For *k* source blocks, the Ideal Soliton distribution *Ω*_is_(*i*) is given by Equation (1):

(1) $$\Omega_{\mathrm{is}}(i)=P(d=i)=\left\{ \begin{array}{ll} 1/k, & \text{if}i=1\\ 1/i(i-1), & \text{if}i=2,...,k \end{array}\right.$$

Let $R=c_{0}\sqrt{k}(k/\delta )$, where *c*_0_ is a suitable constant and 0 < *δ* < 1. The Robust Soliton distribution for *k* source blocks is defined as following. Define:

(2) $$\tau (i)=\left\{ \begin{array}{ll} R/ik & \text{if }i=1,...,k/R\;\\ R\text{ln}(R/\delta )/k & \text{if }i=k/R\\ 0 & \text{if }i=k/R+1,...,k \end{array}\right.$$

The Robust Soliton distribution is given as Equation (3):

(3) $$\Omega_{\mathrm{rs}}=\frac{\tau (i)+\Omega_{\mathrm{is}}(i)}{\sum_{i=1}^{k}\tau (i)+\Omega_{\mathrm{is}}(i)},\text{ for all }i=1,2,...,k\;$$

The decoding process utilizes the Belief Propagation (BP) algorithm [7], which is widely used and very computationally efficient.

#### 4.1.2. LTCDS

**Figure 4.**
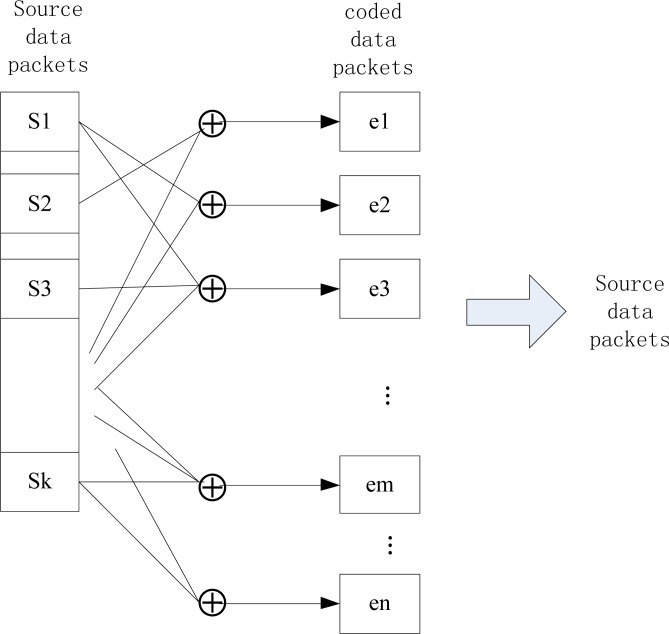
Distributed storage system based on LT code.

The characteristics of the Fountain Codes indicate that they can ensure the persistence of the sensed data of WSN nodes. In order to implement the idea of digital Fountain Codes in WSN systems, Aly *et al.*[6] proposed the LT Codes [7]-based distributed storage algorithm (LTCDS), which can provide persistent data storage solutions with low data redundancy when no geographic or routing information is available. Figure 4 shows the distributed storage model for WSNs based on the LTCDS code.

LTCDS presents pretty good data persistence, with a quite simple coding scheme, and therefore, it can effectively tolerate node failures. However, LTCDS has several shortcomings.

##### (1) Null Storage

In the LTCDS model, the source packets are accepted with probability d(*u*)/*k* by the storage nodes in WSN system, so when the coding process is completed, the overall distribution of the probability of encoded packets stored in the nodes is close, but not equal, to the ideal probability distribution of LT codes. The degree of the coded packets in node *u* is:

(4) $$\begin{array}{ll} \text{Pr}(d^{'}(u)=i) & =\sum_{d(u)=1}^{k}\text{Pr}(d^{'}(u)=i|d(u))\Omega '(d(u))\\ & =\sum_{d(u)=1}^{k}\left(\begin{array}{l} k\\ i \end{array}\right){\left(\frac{d(u)}{k}\right)}^{i}\left(1-\frac{d(u)}{k}\right)\Omega '(d(u)) \end{array}$$

where Ω’ is the degree distribution of the LT codes, which can be an ideal Soliton distribution or Robust Soliton distribution. Such a degree of distribution is close to that of the original LT codes, which helps to maintain the decoding characteristics of LT codes. However, in actual networks, if a node fails to generate enough codes, this node may refuse to store any data packets forwarded to it. Then the null storage phenomenon may appear, because the actual encoding degree of such nodes may become zero, and the storage of this node may become invalid. Null storage phenomena may wreck the ideal degree distribution, which is not conducive to persistence of WSNs.

##### (2) ‘Cliff Effect’

From Equation (4), it can be concluded that the data packets with degree 1 (original data packets without coding with other packets) play a very important role for LTCDS, and they shall directly affect the coding performance. On the other hand, packets with degree 1 are also important for the decoding performance, because they act as basic factors to decode other packets.

**Figure 5.**
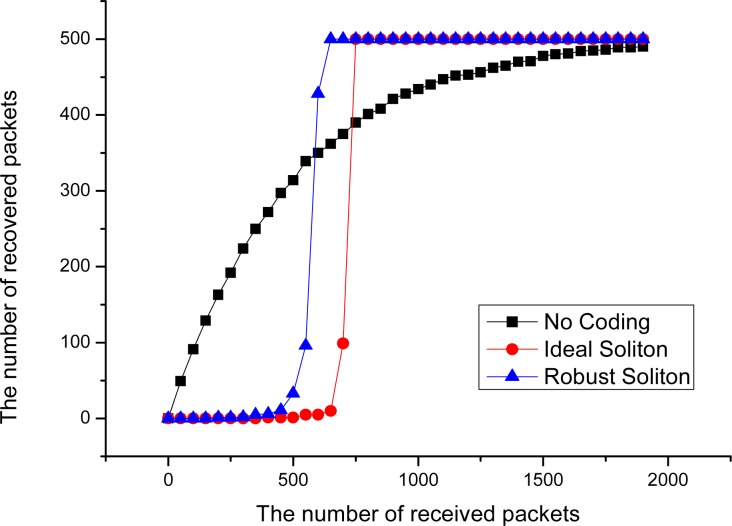
Decoding performance of different models.

Figure 5 shows the decoding performance for LT codes in point-to-point data transmission mode with an Ideal Soliton distribution and Robust Soliton distribution, when the number of source packets *k* = 500. In this figure, it can be seen that the decoding rate is very low before 500 coded packets are received, and the decoding efficiency rapidly increase after more than 700 encoded packets are received. This phenomenon indicates that, due to bad intermediate performance of LT Codes [7], it may cause a serious ‘cliff effect’ [8], and that is to say, source data are hard to recover from the collector node before sufficient encoded packets are received by it. The ‘cliff effect’ can seriously decrease the decoding efficiency in the data collection process. Especially in the early period of data collection, the probability to successfully decode one packet is very small. However, in an extremely harsh WSN environment, the nodes in the network are prone to collapse in a large area at the same time, which may completely destroy the availability of network data before the ‘cliff effect’ period ends. At that time, a large number of packets with high degree may have been collected, but most of these packets may never have any opportunity to be decoded. This phenomenon can seriously decrease the data persistence and collection efficiency of WSN systems.

### 4.2. PLTD-ALPHA Algorithm

A simple backup mechanism is a typical method to improve data persistence of distributed information. With a simple backup mechanism, one node simply forwards source data packets to its neighbors, and the neighbors store this packet or just forward it too. Finally, a source packet can be stored in multiple nodes in the network. If a node fails, and its data packet is lost, the overall data persistence wouldn’t be affected, since other nodes still possess their copies of that packet. However, such a mechanism shall cause serious data redundancy which greatly affects the efficiency of the network. Furthermore, considering the uncertainty of the fault type and location of sensor nodes, a simple backup mechanism can hardly prevent the object data and its simple backup from being destroyed at the same time.

A simple ideal experiment can be designed to illustrate the problem. Suppose there is a point-to-point communication model, and 500 different source packets are continuously transmitted to the receiving end from the transmitting side. With different coding methods, we have the result in Figure 5. From this curve, it can be seen that, without an efficient coding method, the decoding efficiency of the receiving end is satisfactory in the beginning, which is basically close to the case of linear growth. But the decoding efficiency decreases sharply in the latter period; this phenomenon is named the Coupon Collector’s Problem [18]. With the increase of the number of the source data collected, the probability to recover the new source decreases.

Data persistence is enhanced when LT code is introduced to improve the distributed data storage mechanism of a wireless sensor network, but due to the essential coding characteristics of LT codes, in the early decoding period the decoding rate is less than 30%, and the overall decoding performance curve appears as a ‘cliff’ shape. For example, suppose that there are four source data packets *s*_1_, *s*_2_, *s*_3_, *s*_4_, to be encoded with LT codes. The sink node may receive such code set as: *s*_2_, *s*_1_ ⊕ *s*_2_, *s*_2_ ⊕ *s*_3_, *s*_2_ ⊕ *s*_3_ ⊕ *s*_4._ In this situation, if the sink node doesn’t receive *s*_2_, none of other packets can be recovered from this set, which leads to low data decoding rate and affects the performance of the whole system.

Essentially, this phenomenon appears because the strategy with LT codes freely forwards and encodes data out of control, which randomly immingles data packets with different coding degree together in the whole network system. Consequently, in the early decoding period, packets with high degree cannot be decoded due to the lack of sufficient decoding factor with low degree.

**Figure 6.**
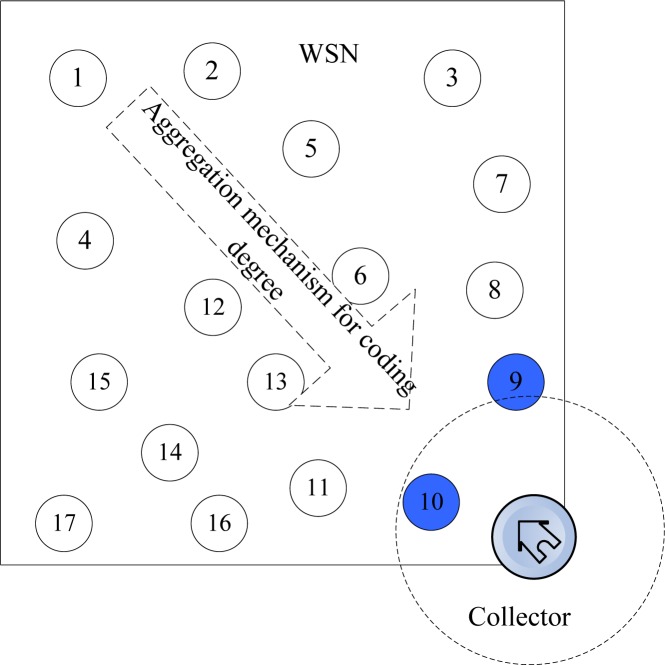
Aggregation mechanisms for coding degree in PLTD-ALPHA.

In this paper, in order to solve the cliff effect problem, the influence of degree distribution strategy to cliff effect is to be observed, and the strategy of optimized persistent data allocation strategy is to be proposed to help submitting data to the sink node in order according to their degree (as in Figure 6). Based on these ideas, we will propose PLTD-ALPHA with predominant decoding performance to improve the data collection performance and decoding efficiency while data persistence is not notably affected. The PLTD-ALPHA algorithm is designed as follows.

#### 4.2.1. Initialization Phase

##### (1) Packet Header Fields Design

**Figure 7.**
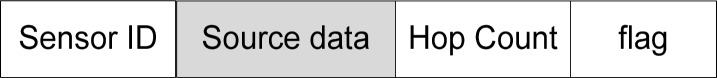
Packet header fields.

Each source node has an *ID_v_*_i_ and sensed data *x_v_*_i_. The node puts its ID into the packet header. As depicted in Figure 7, the packet contains a hop count field *c*(*x_v_*_i_), and a flag indicating whether the data are new or just update of the previous values. According to [6], we set the hop count to *C_n_*log*n* to guarantee that the source packet visits each node in the network at least once, where *C* is a system parameter.

##### (2) Network Constructing with Degree Level

In order to overcome the “cliff effect”, the distribution layout of coded data in WSN systems are expected to present a trend that their degree increases along with some predefined framework, so the persistent data packets can be submitted to the sink node in order, according to their degree level. In the initial network, the default degree level of every node is zero. Before the encoding phrase commences, the collector at the predefined position broadcasts a beacon, and the nodes which receive this beacon automatically adjust its degree level to 1. Then these nodes are regarded as prioritized nodes, which form a network marked with prioritized degree level finally.

##### (3) Nodes Producing Code Degree

Before source nodes disseminate data packets, each node shall decide its object code degree as follows: the prioritized nodes (nodes marked degree level one in advance) set their object code degree as *d*_c_(*u*) = 1. The rest nodes shall draw a random number *d*_c_(*u*) according to the Ideal Soliton distribution *Ω*_is_(*d*).

#### 4.2.2. Extended Degree Level Framework

The “Degree level” constructed in the initialization phase is quite important for PLTD-ALPHA, since this mechanism forms a network marked with prioritized degree level, which remarkably advances the high-speed decoding period so that the “cliff effect” of WSN systems can be greatly alleviated.

However, the nodes in the non-prioritized area still obey the completely random method to select the neighbors and to exchange data. It has been analyzed previously that it is also impractical to adopt routing models to resolve this problem, since in a routing model, some key nodes may have to continuously undertake heavy communication tasks while other nodes have few opportunities to forward messages, which can affect the balance of energy consumption in the WSN and shorten the life cycle of the whole system. Generally, a completely random mechanism is beneficial to the balance of energy consumption in WSN. But if a large-scale WSN system is to be constructed, the nodes far away from the sink node may have low probability to deliver their data to the sink node, so that such data may need long time to reach the sink node, or even never reach the sink before the end of encoding phrase.

Therefore, in order to improve the efficiency of data dissemination, the “degree level framework” is extended in this part, which is fixed after construction, and requires no periodical update process. The basic rule of the extended degree level framework is that each node selects the communication object within its neighbor nodes according to the level list of its neighbor nodes with different probability. The greater the level of a neighbor node is, the lower its selection probability is. The neighbor nodes with equal level shall be selected with an equal probability. The main process of extended “Degree level” framework construction is depicted by the following steps:

1. When the prioritized degree level framework is set up, the nodes which receive this beacon shall automatically adjust their degree level to 1 (as prioritized nodes).
2. Then all the prioritized nodes (nodes with degree 1) activate the second round broadcast. Each node receives these broadcast signals shall check its own level tag, and if its level tag is still 0, it should update the level to 2. Otherwise, if the level tag of a node is no longer 0, there will be no updating, since this node should have been updated as a node of lower level in some earlier round.
3. Then all the nodes with degree 2 shall activate the third round broadcast. Each node receives these broadcast signals shall check its own level tag, and if its level tag is still 0, it should update the level to 3. Otherwise, if the level tag of a node is no longer 0, there will be no updating, since this node should have been updated as a node of lower level in some earlier round.
4. Similarly, all the nodes with degree *i* shall activate the (*i* + 1)th round broadcast. Each node receives these broadcast signals shall check its own level tag, and if its level tag is still 0, it should update the level to *i* + 1. Otherwise, if the level tag of a node is no longer 0, there will be no updating.
5. “Extended degree level framework” construction algorithm ends when all nodes updated.

In the process of data transmission, each node chooses one of its neighbors with different probability. The greater the level of a neighbor node is, the lower its selection probability is. The neighbor nodes with equal level shall be selected with an equal probability. This strategy not only avoids the drawback of completely random, but avoids the high cost due to employing routing technique. Meanwhile it can improve the efficiency that the nodes far away from the sink node to deliver their data to sink.

**Figure 8.**
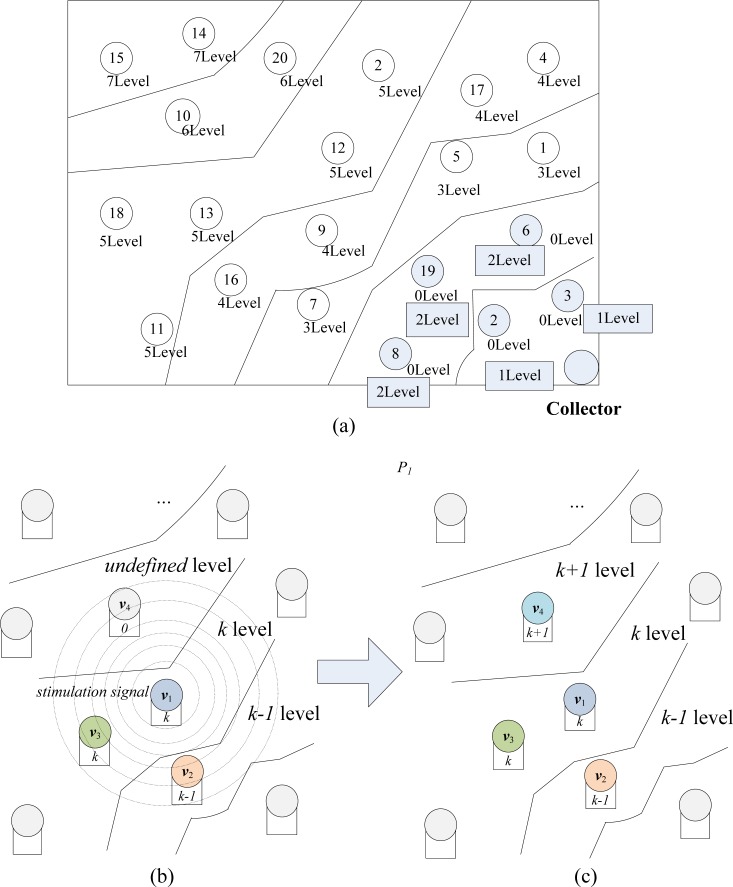
The principle of extended “node level” construction algorithm.

The main process of extended degree level construction is demonstrated in Figure 8. A sample WSN system with full degree level framework is shown in Figure 8(a), and the whole system is divided into seven degree level areas.

In Figure 8(b), a random node *v*_1_ is broadcasting a stimulation signal to update the degree level status of its neighbor nodes *v*_2_, *v*_3_ and *v*_4_. Before being updated, *L*(*v*_2_), the degree level of *v*_2_ is *k* − 1, *L*(*v*_3_) = *k* and *L*(*v*_4_) = 0. Then, when these neighbor nodes capture the stimulation signal, each node shall check its own degree level. In this figure, we can see that the original degree level of *v*_4_ is 0, which implies that it has never been updated. Therefore, as in Figure 8(c), *v*_4_ updates its level *L*(*v*_4_) = *L*(*v*_1_) + 1 = *k* + 1. However, the level of *v*_2_ and *v*_3_ will keep unchanged and they won’t broadcast any stimulation signal after this moment, since they have been updated before this round and broadcasted once. When the nodes begin to disseminate and encode the data packets, one node among *v*_2_, *v*_3_, *v*_4_ will be randomly selected to exchange data with *v*_1_, with different selection probabilities *P*_2_, *P*_3_ and *P*_4_ (*P*_2_ > *P*_3_ > *P*_4_). The construction algorithm of “node level” framework is described as Algorithm 1.

**Algorithm 1.** The full “node level” construction strategy.

Initialization process. (*N* Nodes deployed, collector ready and network topology defined); Data Generation (Each node *i* generates a source data packet *x*_i_); “Node level” framework Initiation. (for each node, *L*(*v*_i_) = 0); Set the level of the collector node as 1; Set *i* = 1; Nodes of level *i* broadcast a stimulation signal to its neighbors; Each node receives these broadcast signals from level *i* checks its own level tag; If its level tag is still 0, its level tag is set to *i* + 1; Else, its level tag keeps unchanged; If any node with level 0 still exists, goto 6; Disseminate and encoding of data packets commences.

#### 4.2.3. Encoding Phase

After establishing the extended degree level framework, the nodes in the WSN system begin to disseminate and encode the source packets. Each source node *v*_i_ sends out its own source packet to another node *u* which is randomly chosen from all its neighbors. When node *u* accepts this source packet the encoding algorithm (Algorithm 2) shall be called to determine whether to update the storage or directly forward the packet. Each node receiving the packet will check the ID and maintain a class of cumulative counter *count*(*i*) to record the total number of different source packets it has received.

When an arbitrary node *v* receives the data *x_v_*_i_ from its neighbor node *u*, it will make decisions as follows:

1. If it is the first time that *x_v_*_i_ visits *v*, the node shall add the *count*(v) by one, and then decide whether to accept or reject the data. If *count*(v) < *k*, the node will accept the data with probability *d*_c_(*v*)/ *k*. If accepted, it will update the storage as $y_{v}^{+}=y_{v}^{-}⊕x_{vi}$. But if *count*(v) = *k*, while at the same time the actual encoding degree still remains zero, then the node will accept the data with probability one. No matter *x_vi_* is accepted or not, the node *v* will decrease the hop count of this packet by one as *c*(*x_v_*_i_) = *c*(*x_v_*_i_) − 1, and puts this packet into its forwarding queue.
2. If *x_v_*_i_ has visited *v* before, the node does not determine whether to accept or not, but directly decreases the hop count by one as *c*(*x_v_*_i_) = *c*(*x_v_*_i_) − 1, and simply puts *x_v_*_i_ into its forward queue.
3. Inside the forwarding queue, node *v* will check if the hop count of a packet is zero. If not, this packet will be forward to one of its neighbor node. Otherwise, this packet will be discarded.

**Algorithm 2.** Encoding algorithm.

**Algorithm of Encoding**
While node *v* receives *Packet(x_vi_)*, do if *v* receives *x_vi_* for the first time, then *count(v) ++*; if *count(v)* < *k*, then {*temp* = rand(1); if(*temp* < *d_c_(v)/k*), then $\left\{y_{v}^{+}=y_{v}^{-}⊕x_{\mathrm{vi}};c\left(x_{\mathrm{vi}}\right)=c\left(x_{\mathrm{vi}}\right)-1;\right\}$ else if (count(v)==*k* && the real encoding degree == 0), then $\left\{y_{v}^{+}=y_{v}^{-}⊕x_{\mathrm{vi}};c\left(x_{\mathrm{vi}}\right)=c\left(x_{\mathrm{vi}}\right)-1;\right\}$ Put *x_vi_* into *v’s* forward queue; else if *v* receives *x_vi_* not for the first time, then { *c*(*x_vi_*) = *c*(*x_vi_*) − 1; Put *x_vi_* into *v’s* forward queue;} if *c*(*x_vi_*) =*=* 1 Discard *x_vi_*; else forward (*x_vi_*).

**Figure 9.**
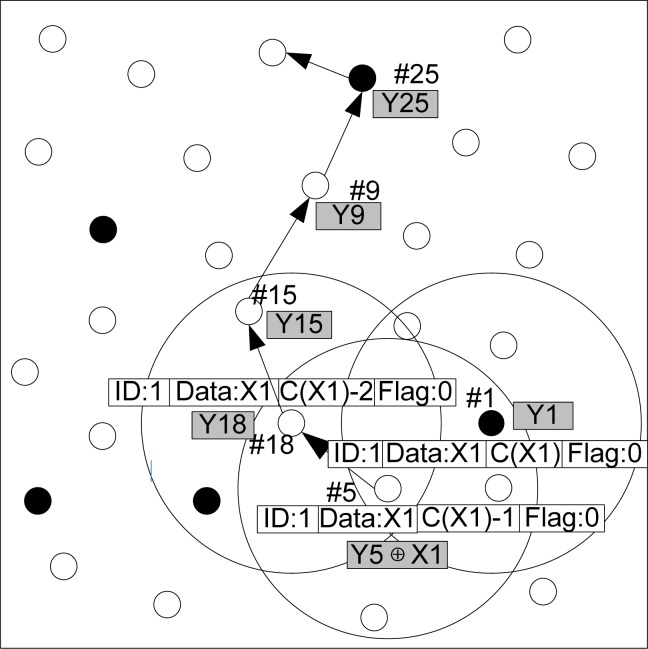
Example of a data packet forwarding in the network.

Figure 9 presents an example of packet dissemination and encoding phase in the WSN network. The source node with *ID* 1 generates a source data packet *x*_1_ with hop count *c*(*x*_1_). It randomly chooses a neighbor node (*ID* = 5) and forwards *x*_1_ to it, while the hop count of *x*_1_ is decreased by one. This node accepts *x*_1_ according to the algorithm described in Algorithm 2. If *x*_1_ is accepted, the storage content of this packet shall be updated as *y*_5 =_*y*_5_ ⊕ *x*_1_. Then this node continues to send the encoded data to a neighbor node with *ID* 18 randomly, and the hop count of this source data packet is reduced by one again. If the node with ID = 8 does not need to encode this packet, its storage still remains *y*_18_. This packet shall be forwarded until the hop count is reduced to zero, and then this packet is to be discarded forever.

#### 4.2.4. Storage Phase

If the hop count of a packet is zero, the node possesses it will stop forwarding it. When all packets in the WSN system stop been forwarded, encoding phase ends and the storage phase commences. The nodes do not update their storage any more. In the storage phase, each node possesses one packet with encoded data in its buffer.

#### 4.2.5. Decoding Phase

After encoding phase, the mobile data collector gets into the network from a certain entry of the network and visits sensor nodes following a certain path. The collector can recover all source data after gathering a number of encoded packets.

### 4.3. Data Storage for Updating

As a supplementary description of the PLTD system [32], the data updating strategy for PLTD-ALPHA is described as follows:

1. Suppose the data packets *y*_1_, *y*_2_,…, *y_n_* have already been stored in the current network nodes. If the sensor node *u* is going to possess the sensed data *x_u_*, its data content can be represented as *packet_u_* = (*ID_u_*, *x_u_*, *c*(*x_u_*), *flag*). Then the *flag* bit in the packet is set to 0.
2. It can be seen from the previous description that, the data packets are distributed into the whole network with random walk manner. Suppose that *x*′*_u_* is the new data packet in node *u*. Node *u* shall firstly update its own data packets, and that is to say, the new data are XORed together with the original data in node *u. packet_u_= (ID_u_*, *x_u_* ⊕ *x*′*_u_*, *c(x_u_)*, *flag*), and then the *flag* bit in the packet is set to 1.
3. Each data packet encodes and stores the *flag* bits required by its owner node to verify the data packet. If *flag* = 1, the encoded packets stored in this node shall be updated. If a node *v* detect a data packet updated, and the ID number of this data packet is right in the ID number array recorded by current node, node *v* shall update its storage unit as $y_{v}^{+}=y_{v}^{-}⊕x_{u}^{'}⊕x_{u}$.

### 4.4. PLTD-ALPHA Analysis

According to the degree of distribution characteristics of a network with a PLTD-ALPHA mechanism, when network coding is completed, the mobile collector gets into the network. In this scenario, in the early period, the collector visits the nodes around it (in the prioritized area), so it can quickly gather the encode packets with low coding degree. After this period, when the collector passes through the boundary between the prioritized and non-prioritized area, it begins to collect other encoded packets generated based on the degree distribution of LT codes, such as the Ideal Soliton distribution.

Supposing that the collector gathers *m* encoded packets around it, the distribution of these packets can greatly affect the decoding speed. Considering the previous analysis, with PLTD-ALPHA, the high-speed decoding period can begin much earlier, and the “cliff effect” can be greatly relieved. In such WSN system, *m* << *n*, and *m* < *k* are workable, and the prioritized nodes make up only a small proportion of the total number of nodes in the network, which doesn’t bring a great deal of redundancy. To evaluate the performance of PLTD-ALPHA when the prioritized nodes fail, the worst case can be considered. That is to say, we consider the situation that all sensor nodes fail before the collector gets into the network. All nodes generate initial code degree according to the distribution of LT codes. In this case, the performance of PLTD-ALPHA is greatly reduced, but it is still not worse than that the performance of classical LTCDS.

Since the main purpose of PLTD-ALPHA algorithm is to improve the data persistence of WSN and the efficiency of data recovery under extreme or disaster scene, it’s critical to analyses the performance of PLTD-ALPHA algorithm in all kinds of node failure scenarios. Two types of typical disaster scenarios are considered in this work.

The first type is for some uncertain and burst disaster scenario. Within the overall network coverage area *Area*_all_, node failures may concentrate in some local areas. The performance in two kind of situation is to be evaluated:

1. The first kind is that all failures are concentrated in a non-prior coding degree region *Area*_normal_. Since in the encoding process, the raw data in this region has already been distributed to and stored in the entire coding region with redundancy, and therefore its data can still be decoded in conjunction with other encoded data packets. The whole system still exhibits efficient decoding and recovery performance, since the nodes in the priority region are unaffected.
2. If the failures concentrate in a prior coding degree region *Area*_prior_, it is a rather extreme case. In this case, the data in the whole prior coding degree region *Area*_prior_ are all invalidated. But their information has been encoded into the data packets in the nodes of *Area*_normal_ in the previous encoding process. Therefore, the availability of data is still assured, but the decoding and recovery performance decrease to the same level as the original LT code because all node in *Area*_prior_ are unavailable.

The second type is for the situation that sensor nodes fail evenly in the entire coverage region *Area*_all_ of WSN network. The results and data of such analysis are to be demonstrated and discussed in the experiment and result section.

## 5. Results and Performance

In the following sections, a series of experiments are to be designed to testify the performance of PLTD-ALPHA algorithm. In these experiments, each is executed 100 times, and the average value is adopted as the result, the hop count coefficient is set to *C* = 3. The location of the data collector is (0, 0). Collection phase commence after the encoding phase is completed.

### 5.1. Data Collecting in Random Networks

**Figure 10.**
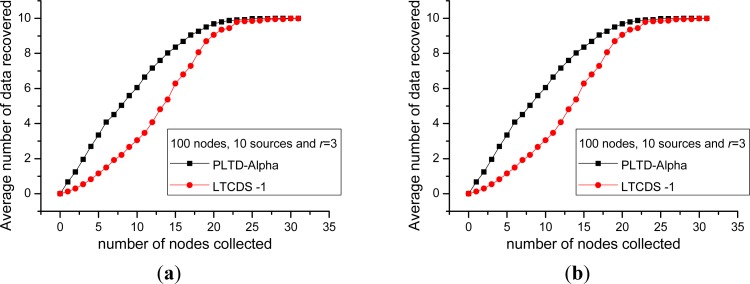
Data recovery performance in small scale WSN system. (**a**) *n* = 100, *k* = 10 (**b**) *n* = 100, *k* = 20.

**Figure 11.**
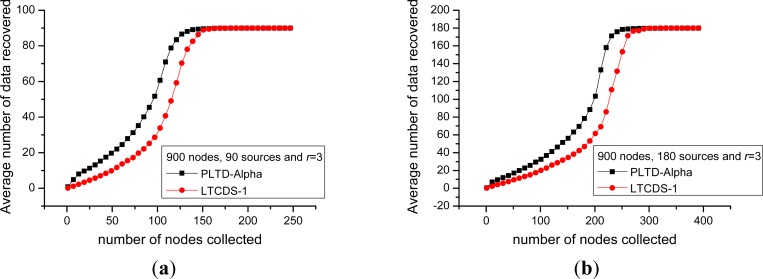
Data recovery performance in large scale WSN system. (**a**) *n* = 900, *k* = 90, (**b**) *n* = 900, *k* = 180.

Firstly, we compare the data collection efficiency of PLTD-ALPHA and LTCDS-1. Figure 10 shows the decoding performance with a small number of nodes and sources. The network is deployed in a 10 × 10 field, the total number of nodes is *n* = 100, the number of source nodes are *k* = 10 and *k* = 20 respectively in Figure 10(a, b). In Figure 11, the number of nodes is increased to *n* = 900, *k* = 90 and 180, while the nodes are deployed in a 30 × 30 area, but the network density is kept unchanged. In Figures 10 and 11, *X* axis indicates the average number of encoded packets collected by a data collector while *Y* axis represents the average number of source data decoded from the collected data. The communication radius of nodes is set as *r* = 3.

Figures 10 and 11 show that PLTD-ALPHA achieves higher data collection efficiency than LTCDS-1, especially when the number of collected data is small (the early period of the “cliff effect”). PLTD-ALPHA presents higher decoding rate and effectively restrains the ‘cliff effect’ of LTCDS-1 codes while data persistence is not notably affected (since the data distribution is not notably affected).

Meanwhile, Figures 10 and 11 also show that, with PLTD-Alpha or LTCDS-1, all data can be recovered after a certain period of time. That is to say, when the aggregation mechanism for coding degree in PLTD-ALPHA is introduced, the data persistence of the sensor network is not significantly affected.

### 5.2. Data Collecting in a Disaster Network

**Figure 12.**
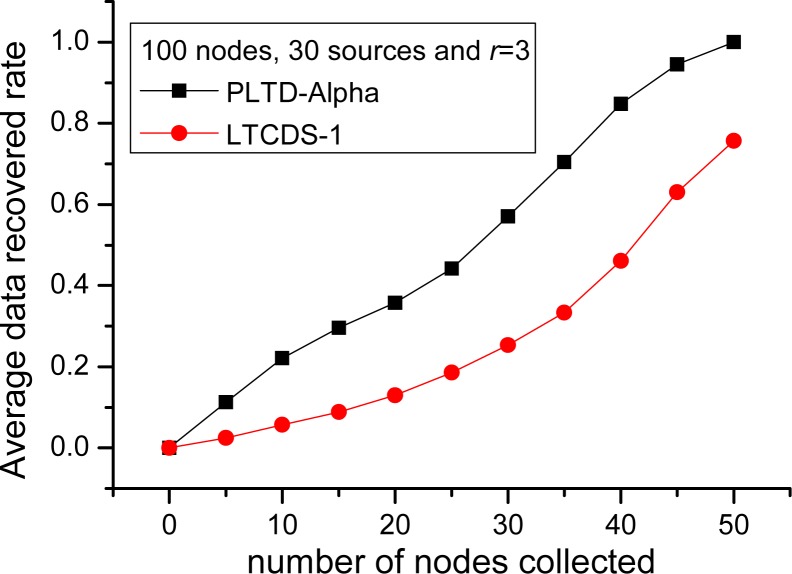
Data recovery performance in a disaster network with *n* = 100, *k* = 30.

**Figure 13.**
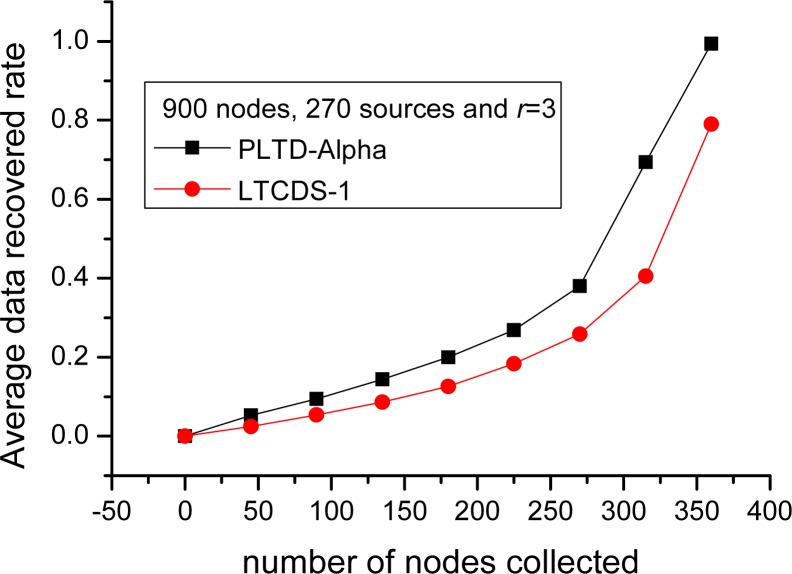
Data recovery performance in a disaster network with *n* = 900, *k* = 270.

Sensor networks can be employed in emergency or disaster scenarios where the WSN systems are subject to node failures. PLTD-ALPHA has an advantage for data collection in disaster-prone areas. For example, a subset of the sensor nodes may be destroyed and stop working while an earthquake happens, since the sensor nodes deployed in the region affected by the earthquake may get ruined. In this part, the performance of PLTD-ALPHA in a disaster network is evaluated, with the result shown in Figures 12 and 13. In Figure 12 the network consists of 100 nodes and among them 30 are source nodes. We assume that the collector has visited 50 nodes before the network collapse and it cannot continue to collect any more data. Figure 12 shows that more source data can be recovered with PLTD-ALPHA, but the data recovery rate with LTCDS-1 is only 0.75, which indicates that PLTD-ALPHA performs much better in the sudden disaster scenario than LTCDS-1.

Similarly, as shown in Figure 13, when the number of nodes is increased to *n* = 900 and *k* = 270, the performance of PLTD-ALPHA is also better than LTCDS-1. Supposing that 360 data packets have been collected when the network collapse, the average data recovery rate of PLTD-ALPHA is about 0.995, which is close to 1. However, the recovery rate is only 0.78 with LTCDS-1. That is to say, the performance of PLTD-ALPHA in large network is also better.

### 5.3. Performance with Different Network Connectivity

**Figure 14.**
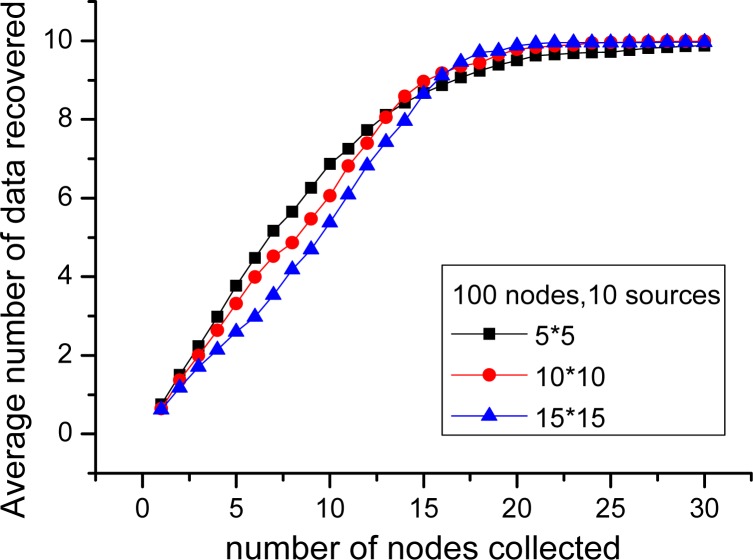
Data recovered with different network density.

**Figure 15.**
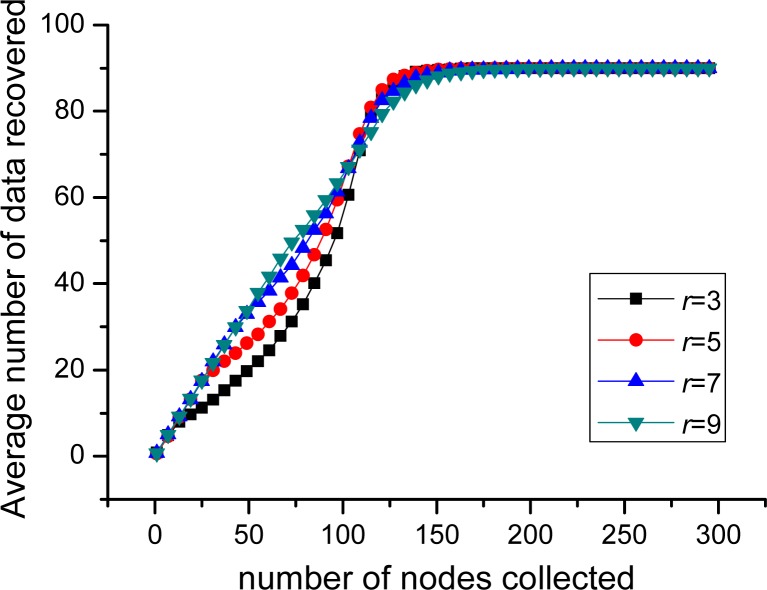
Data recovered in network with different communication radius.

In this part, the performance of PLTD-ALPHA with different network connectivity is to be investigated by changing the network density and the communication radius r. First, for the scenario of 100 nodes and 10 sources, *r* = 3, sensor nodes are respectively deployed in 5 × 5, 10 × 10 and 15 × 15 area. As shown in Figure 14, during the phase of collecting 1∼15 nodes, the average number of source data decoded in 5 × 5 is more than in 15 × 15, while later it is slightly less than in 15 × 15. Then, we change the communication radius of nodes r. In Figure 15, *n* = 900 and *k* = 90 and the nodes are randomly distributed in 30 × 30 area. We switch *r* = 3, 5, 7, 9, but the result curves indicate that the performance is similar as Figure 14. Therefore, in networks with high connectivity, the average decoding ratio is higher while in the low connective network there will be a slight performance declining.

This phenomenon happens because in the network with better connectivity, more nodes can receive the beacon signal broadcasted by the collector. Accordingly, the number of nodes with priority increases. These nodes can improve the collecting performance in the early decoding period but more nodes with priority can also lead to serious data redundancy and communication redundancy. However, such impact is not remarkable, because in an actual network, the nodes with priority only take a small portion in the network.

### 5.4. Complexity Analysis

For PLTD-ALPHA, as a prioritized distributed data storage and dissemination algorithm, the complexity of its data dissemination process and the establishment of prioritized area and the extended degree level framework can greatly affect the performance of data recovery of WSN systems. Since there are *n* sensor nodes randomly deployed in the network, the problem scale can be regarded as *n*.

#### 5.4.1. Complexity of Establishment of Prioritized Area and Extended Degree Level Framework

Both prioritized area and the extended degree level framework construct the whole coding degree distribution level of PLTD-ALPHA. Data in sensor network nodes are expected to present a trend that their degree distribution increases along with such degree level predefined, and then the persistent data packets can be submitted to the sink node according to its degree in order, so that the “Cliff Effect” can be notably relieved.

##### (1) Prioritized Area Establishment

In this process, the default degree level of each node is zero. The collector at the predefined position broadcasts a beacon, and the nodes which receive this beacon automatically adjust their degree level to 1. Then these nodes are regarded as prioritized nodes. Since each node in the prioritized area receive such beacon signal and update its degree level for only once, and in this model, the prioritized nodes make up only a small proportion of all nodes in the WSN system, the number of key operations can be calculated as Equation (5).

(5) $$f_{p}(n)=p_{1}*n;\;\;\;\;\;p_{1}\ll 1$$

##### (2) Extended Degree level Framework Establishment

With a random walk model, in order to further improve the efficiency of data dispersion, the degree level framework is extended into an extended degree level strategy. In this process, all the nodes with degree *i* shall activate the (*i* + 1)th round broadcast. Each node receives these broadcast signals shall check its own level tag. If its level tag is still 0, it should update the level to *i* + 1. Otherwise, there is no updating.

So, it can be concluded that each node out of the prioritized area can receive the beacon signal for several times, but its level tag can only be updated once, from 0 to a non-zero degree level. The number of updating operations is *f*_level1_(n) = (1 − *p*_1_)*n. Accordingly, after such updating, each node possesses an unique non-zero degree level, and can activates a broadcast beacon signal once for the next level nodes. It is obviously that this process is much different than a flooding network broadcast procedure, and the number of broadcasting operations is *f*_level2_(n) = (1 − *p*_1_)*n.

##### (3) Complexity of Establishment of Coding Degree

Given all that, the complexity of this part can be evaluated by Equation (6):

(6) $$\begin{array}{l} O(f_{\text{deg}\mathrm{ree}}(n))=O(f_{p}(n)+f_{\text{level}1}\text{(n)}+f_{\text{level}2}\text{(n)})=O(p_{1}*n+2*(1-p_{1})*n)=O(n);\\ p_{1}<<1 \end{array}$$

#### 5.4.2. Complexity of Data Dissemination

In the dissemination and encoding process, each source node *v*_i_ sends out its own source packet to another node *u* which is randomly chosen from all the neighbors of *v*_i_. When node *u* accepts this source packet the encoding algorithm shall be called to determine whether to update the storage or directly forward the packet. When an arbitrary node *v* receives the data *x_v_*_i_ from its neighbor node *u*, no matter *x_vi_* is accepted or not, the node *v* will decrease the hop count of this packet by one as *c*(*x_v_*_i_) = *c*(*x_v_*_i_) − 1. Then, node *v* will check the hop count of a packet, and if it is zero, this packet will be discarded.

Since in this WSN system, the degree distribution of most sensor nodes (except the nodes in the prioritized area) is arranged according to Ideal Soliton distribution *Ω*_is_(*d*), the maximum degree level in each node is less than *n*. Therefore, *c*(*x_v_*_i_) is in the same order with *n*. Accordingly, the number of key operations in node *x_vi_* is *f*_d−i_(n) = *c*(*x_v_*_i_)+ *c_e_*(*x_v_*_i_). Therein, *c_e_*(*x_v_*_i_) is the number of encoding operation for data packet *x_vi_*, which is less than *x_vi_*, since not every data forwarding process give rise to an encoding operation. On the other hand, the whole data dissemination period is a parallel process, so the global complexity can be estimated according to that of a single node *x_vi_* as Equation (7):

(7) $$O(f_{d}(n))=O(f_{d-i}(n))=O(C(x_{\mathrm{vi}})+C_{e}(x_{\mathrm{vi}}))<O(2*C(x_{\mathrm{vi}}))=O(n);\;\;\;\;\;\;p_{1}<<1$$

#### 5.4.3. Analysis of Extreme Situations

In order to verify the performance of PLTD-ALPHA in different situations, a series of experiments has already been designed and performed. In Figures 10 and 11, the performance of PLTD-ALPHA in WSN systems of different scale is evaluated while data density is kept unchanged. Figure 10 shows the decoding performance with small number of nodes and sources, while Figure 11 demonstrates that of a larger scale WSN system. Figures 12 and 13 also demonstrate the performance in WSN systems of different scale, but in a disaster scenario. The performance in WSNs of different density and communication radius is also evaluated in Figures 14 and 15.

As for more extreme situations, the minimum scale case is a WSN with only one source node, and the unique source data packet generated by it won’t have any other object packets to encode. The maximum scale case is WSN system in which all nodes generate source data. In such system, there is no redundant storage node available to store the encoded data.

The WSN with minimum connectivity can be regarded as a point-to-point system. In this case, all data has only one route to disseminate its data, which is degenerated as a routing network model, so PLTD-ALPHA can’t improve its performance. On the other hand, the WSN with maximum connectivity implies that every node in the network can receive the beacon signal from the collector node, so that every nodes of the network are included in the prioritized area. In such case, every node keeps degree 1, and there are not any network coding factors in such WSN system.

## 6. Conclusions

For wireless sensor network systems deployed in a disaster scenario, sensor nodes are prone to fail in a short period, which can seriously affect the availability of source data. Data persistence is a key factor to measure the reliability of such system. Traditional distributed coding strategy such as LT codes can greatly improve network data persistence but may cause a serious ‘cliff effect’ in the decoding process.

In this paper, the influence of degree distribution strategy on the ‘cliff effect’ is observed, and the characteristics of behavior and data distribution in homogeneous WSN systems are established. A class of prioritized coding degree distribution strategy is analyzed, and LT Codes-based prioritized distributed data storage and dissemination algorithm PLTD-ALPHA is presented to achieve better data persistence and recovery performance. With PLTD-ALPHA, the data in sensor network nodes present a trend that their degree distribution increases along with the degree level predefined, and then the persistent data packets can be submitted in order to the sink node according to its degree.

The performance of PLTD-ALPHA is evaluated through various simulations in the networks of different scale. Experiment results show that PLTD-ALPHA can greatly improves the data collection performance and decoding efficiency while data persistence is not notably affected. Meanwhile, the performance of PLTD-ALPHA with different network connectivity is also tested, and the result data also indicates that PLTD-ALPHA has dominant performance.

The shortcoming of PLTD-ALPHA is that limited global information about the sensor network is required to construct the degree level configuration, which increases the difficulty for the deployment of zero-configuration networks. In summary, PLTD-ALPHA provides a good solution to improve data persistence and collecting efficiency. In our future work, we will focus on the self-adaptation feature of PLTD, in order to further improve the performance and reduce the dependency on network configuration information. On the other hand, we will also try to extend the basic idea of PLTD-ALPHA for the practical sensor nodes with much more storage and communication resources.
